# Unidirectional versus direction-selectable traction device in gastric endoscopic submucosal dissection: a randomized controlled trial

**DOI:** 10.1007/s00464-025-12102-8

**Published:** 2025-08-20

**Authors:** Mitsuru Nagata, Masayuki Namiki, Tomoaki Fujikawa, Hiromi Munakata

**Affiliations:** 1 Department of Endoscopy, Shonan Fujisawa Tokushukai Hospital, 1-5-1, Tsujidokandai, Fujisawa, Kanagawa Japan; 2 Center for Digestive and Hepato-Biliary-Pancreatic Disease, Shonan Fujisawa Tokushukai Hospital, Fujisawa, Japan

**Keywords:** Endoscopic submucosal dissection, ESD, Traction-assisted ESD, Traction direction, Vertical traction, Traction direction selectivity

## Abstract

**Background:**

The effects of traction direction in traction-assisted gastric endoscopic submucosal dissection (ESD) are underexplored. The clip-with-line (CWL) and spring-and-loop with clip (SLC) are unidirectional and direction-selectable traction devices, respectively. This study compared the procedure-related outcomes of CWL-assisted ESD (CWL-ESD) and SLC-assisted ESD (SLC-ESD) for superficial gastric neoplasms (SGNs).

**Methods:**

This single-center randomized controlled trial included patients with SGNs who were randomly assigned to undergo CWL-ESD or SLC-ESD performed by an expert. The traction direction was classified as proximal, diagonally proximal, vertical, diagonally distal, or distal. Vertical traction (VT) was selected for SLC-ESD using the direction-selectable traction function. The primary endpoint was the median ESD time. The secondary endpoints included dissection speed. Multiple regression analysis was used to identify factors affecting ESD time.

**Results:**

Overall, 105 patients who underwent SLC-ESD (*n* = 52) or CWL-ESD (*n* = 53) between August 2020 and April 2023 were included in the analysis. The median ESD time was significantly shorter in the SLC-ESD group than in the CWL-ESD group (26.0 vs. 40.5 min; *P* = 0.015). The median dissection speed was significantly faster in the SLC-ESD group than in the CWL-ESD group (24.9 vs. 18.2 mm^2^/min; *P* = 0.001). The traction direction significantly differed between the groups (*P* < 0.001), as VT was selected in all cases in the SLC-ESD group, compared with that in 11.3% of cases in the CWL-ESD group. Multiple regression analysis revealed that VT was independently associated with a shorter ESD time (*P* < 0.001). The complete resection rate did not differ between the groups (98.1% vs. 96.2%; *P* = 1.000). Adverse event rates were not different between the groups.

**Conclusions:**

A direction-selectable traction device may be more effective than a unidirectional traction device in gastric ESD.

**Graphical abstract:**

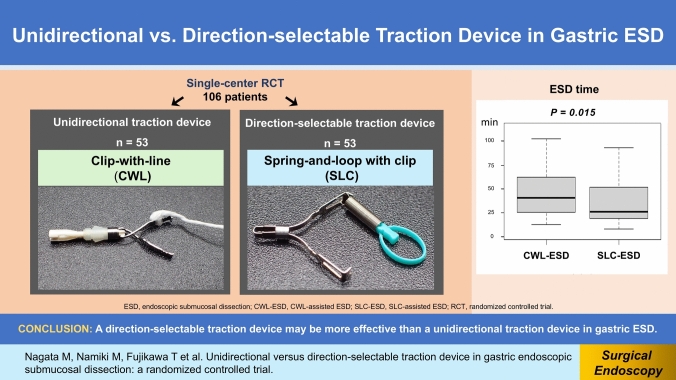

**Supplementary Information:**

The online version contains supplementary material available at 10.1007/s00464-025-12102-8.

Endoscopic submucosal dissection (ESD) is a minimally invasive treatment for superficial gastrointestinal neoplasms. However, gastric ESD is not universally performed because of its technical difficulty, which often results in prolonged procedural times [1, 2]. Although various traction devices have been developed to address these challenges, the selectivity of the traction direction differs among them. For example, the clip-with-line (CWL) is a unidirectional device [3, 4], whereas the spring-and-loop with clip (SLC; Sakamoto-Osada clip, Zeon Medical, Tokyo, Japan) is a direction-selectable device [5]. The traction direction of the CWL in the stomach varies depending on the location of the lesion, as the direction in which the line is pulled is limited to the cardia. In contrast, the SLC allows for controlled traction direction regardless of the lesion location.

Although the impact of the traction direction in traction-assisted gastric ESD has not been adequately investigated, several studies have suggested that the traction direction influences treatment efficacy. A multicenter randomized controlled trial (RCT) found no significant difference in gastric ESD time between CWL-assisted ESD (CWL-ESD) and conventional ESD (C-ESD) [6]. Conversely, a single-center RCT reported that SLC-assisted ESD (SLC-ESD), which applies vertical traction (VT) against the gastric wall, reduced the gastric ESD time compared with C-ESD [7]. These results imply that traction direction affects treatment efficacy and that VT is the optimal direction in gastric ESD.

In a retrospective study using propensity score matching analysis, SLC-ESD with VT had a shorter gastric ESD time than CWL-ESD [8]. However, such research is potentially subject to bias introduced by unobserved differences. We hypothesized that VT is the optimal direction in gastric ESD and that SLC-ESD with VT has a shorter ESD time than CWL-ESD. Therefore, this RCT aimed to compare the procedure-related outcomes between CWL-ESD and SLC-ESD with VT among patients with superficial gastric neoplasms (SGNs).

## Materials and methods

### Study design and study population

In this prospective, single-center (Shonan Fujisawa Tokushukai Hospital, Kanagawa, Japan), parallel two-arm (1:1 allocation ratio), open-label RCT, patients were randomly assigned to the SLC-ESD or CWL-ESD group using computed-based block randomization with a block size of 4, without stratification. Randomization was performed at the point of study enrollment, immediately following the confirmation of participant eligibility and the acquisition of written informed consent. The Mirai Iryo Research Center (Tokyo, Japan) managed patient randomization and data collection, ensuring that the research team had no access to the randomization codes. This trial was approved by the institutional review board of Mirai Iryo Research Center and registered with the University Hospital Medical Information Network (registration number: UMIN000041385) on August 11, 2020. The manuscript conformed to the Consolidated Standards of Reporting Trials (CONSORT) 2010.

Patients who met the following criteria were included: a histopathologically confirmed diagnosis of gastric adenoma or carcinoma, age between 20 and 94 years, no history of gastrectomy or gastric tube reconstruction for esophageal cancer, an Eastern Cooperative Oncology Group performance status of 0–2, provision of written informed consent, and carcinoma with a low likelihood of lymph node metastasis as defined by Japanese guidelines [9]. The following carcinomas were included: clinically intramucosal (cT1a) differentiated carcinomas of any size without ulceration (scar); cT1a differentiated carcinomas of ≤ 30 mm in size with ulceration (scar); and cT1a undifferentiated carcinomas of ≤ 20 mm in size without ulceration (scar). We excluded patients who met the following criteria: pregnancy, potential pregnancy, or lactation in women; prior enrollment in this study; and those deemed ineligible by the investigator for specific reasons.

### Endpoints

The primary endpoint was the ESD time. Secondary endpoints included dissection speed, en bloc resection, intraoperative perforation, traction device-related factors (traction direction, attachment time, number of applications, reattachment, slip-off, traction device-related damage to the specimen, traction device breakage, and successful removal of the anchor clip in SLC-ESD), ESD time based on lesion location (upper and middle thirds vs. lower third), and lesion size (≤ 20 vs. > 20 mm).

### Definitions

ESD time (min) was defined as the time from the first injection to the completion of submucosal dissection, including the time required to attach the traction device. The dissection speed (mm^2^/min) was calculated as the specimen area divided by the ESD time. The length (mm) of the resected specimen was measured after it was pinned to a board. The specimen area (mm^2^) was calculated using the following ellipse formula: specimen area = (shorter axis length)/2 × (longer axis length)/2 × 3.14. The lesion location (i.e., upper, middle, or lower third of the stomach) and lesion position (i.e., greater curvature, lesser curvature, posterior wall, or anterior wall of the stomach) were defined according to Japanese classification [10]. Complete resection was defined as en bloc resection with a negative margin. Intraoperative perforation was diagnosed via endoscopy. Post-ESD bleeding was defined as bleeding necessitating endoscopic hemostasis. The degree of submucosal fibrosis was classified as follows: F0, absent; F1, mild; and F2, severe [11]. The lesion was considered to be on the gravity side when it was located on the side where fluid accumulation occurred.

Regarding specific traction device-related factors, the SLC attachment time was defined as the time from inserting the clip applicator into the accessory channel to completing the anchoring of the loop. CWL attachment time was defined as the duration from the start of endoscope withdrawal to the completion of CWL attachment to the lesion. If traction device reattachment was performed, the time required was included in the traction device attachment time. Traction device-related damage to the specimen was defined as any tear or split of the specimen caused by traction force. Traction device breakage was defined as the line breaking in the CWL and the spring becoming overstretched and losing elasticity in the SLC. In SLC-ESD, removal of the anchor clip was considered successful when it was pulled from the gastric wall and extracted from the body.

**Fig. 1:**
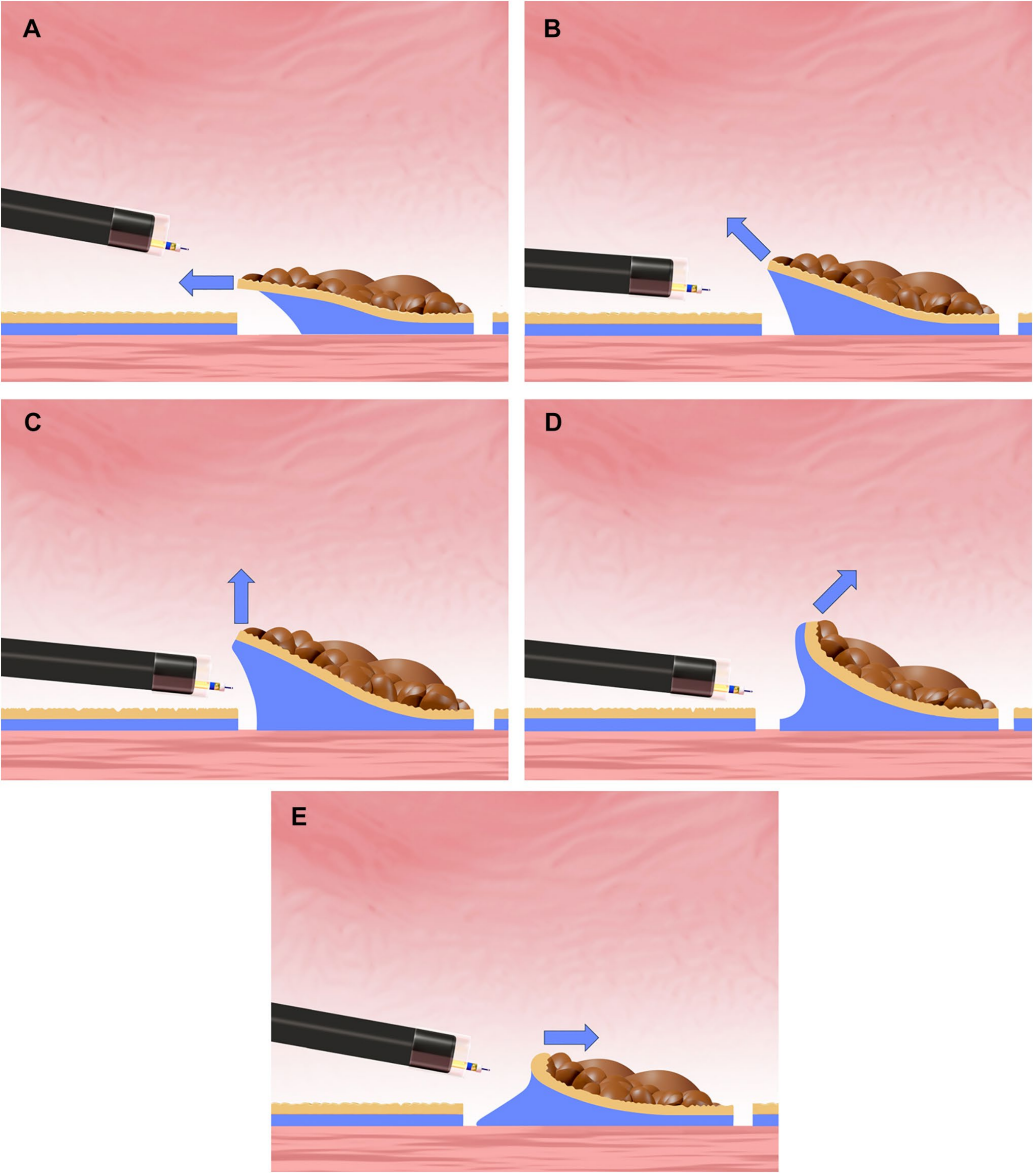
Classification of traction directions. **A** Proximal traction. **B** Diagonally proximal traction. **C** Vertical traction. **D** Diagonally distal traction. **E** Distal traction

Based on endoscopic observations of the spatial relationships among the traction device material (the line component of the CWL or the spring component of the SLC), endoscope tip, and gastric wall, traction direction was classified as proximal, diagonally proximal, vertical, diagonally distal, and distal (Fig. 1) [12].

### Gastric ESD perioperative settings

The patients were hospitalized and treated under intravenous sedation. To eliminate bias related to variations in ESD skills among endoscopists, all procedures were performed by a single endoscopist (M.N.) who had performed more than 1000 ESD procedures, including > 90 SLC-ESD procedures and > 70 CWL-ESD procedures, before the beginning of the trial. A single-channel endoscope with a water supply function (GIF-Q260J; Olympus, Tokyo, Japan) and a straight transparent hood (D-201-11804; Olympus) was used for all ESD procedures. A multibending endoscope (GIF-2TQ260M; Olympus) was used only when it was impossible to approach the lesion with the GIF-Q260J endoscope [13]. Carbon dioxide insufflation was performed to extend the stomach. A straight-needle-type electrosurgical knife without an injection function (KD-650L, DualKnife; Olympus) and hemostatic forceps (FD-412LR, Coagrasper G; Olympus) were used with an electrosurgical generator (VIO300D; ERBE Elektromedizin GmbH, Tübingen, Germany), and a 1:1 mixture of 0.4% hyaluronic acid (MucoUp; Boston Scientific, Marlborough, MA, USA) and saline solution was injected.

### Traction-assisted ESD procedure (Supplementary Video 1)

**Fig. 2:**
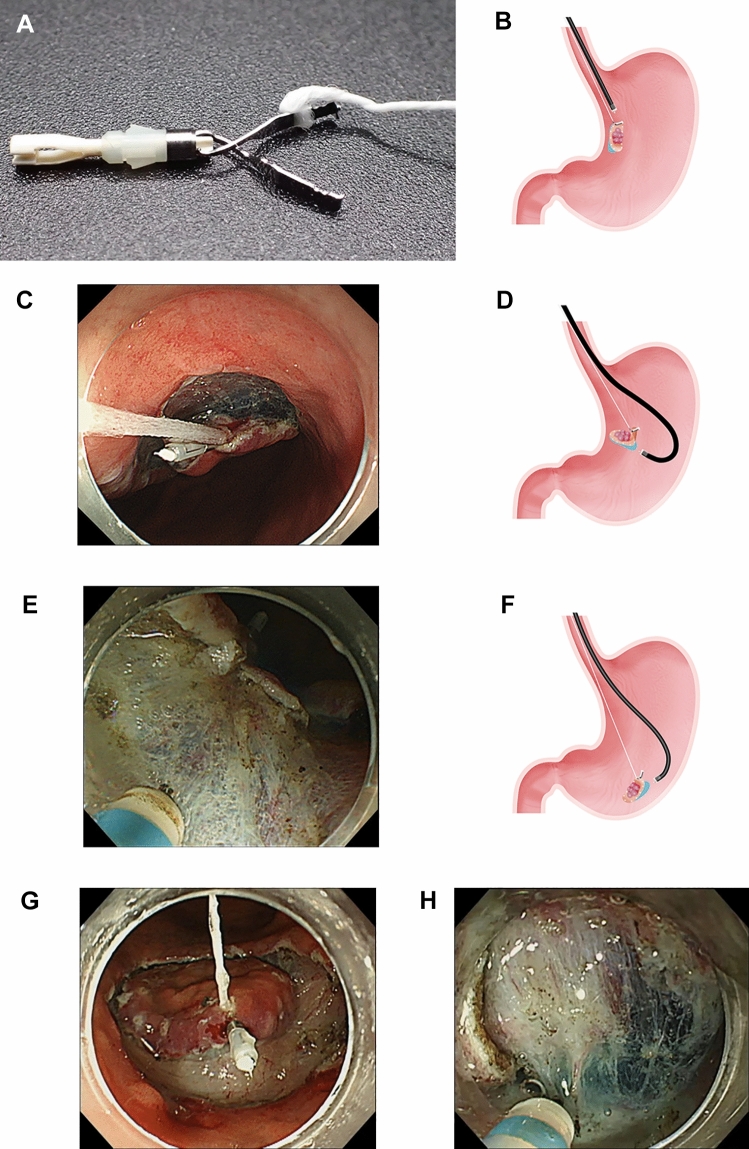
Variation of traction direction depending on the location of the lesion in clip-with-line (CWL)-assisted endoscopic submucosal dissection. **A** A CWL can be handmade by tying commercially available dental floss to the arm part of the clip. **B** Proximal traction. The forward endoscopic position allows for proximal or diagonally proximal traction. **C** Proximal traction can cause the mucosal flap to fall toward the endoscope tip, impeding access to the submucosa. **D** Diagonally distal traction. The retroflexed endoscopic position permits diagonally distal or distal traction. **E** Diagonally distal traction might cause the dissection plane to fall distally as the dissection advances, making the tension on the dissection plane ineffective. **F** Vertical traction. This direction can be obtained if the lesion is located along the greater curvature. **G** Vertical traction. **H** Vertical traction can expose the submucosa and provide effective tension on the dissection plane

CWL-ESD was performed as previously reported (Fig. 2A–H) [14]. After the circumferential mucosal incision, the easier endoscopic position to perform submucosal dissection between the forward and retroflexed endoscopic positions was selected. After determining the endoscopic position, the endoscope was withdrawn, and the clip applicator was passed through the endoscope’s accessory channel. The CWL, featuring a clip (HX-610-090; Olympus) with commercially available waxed nylon dental floss tied to its arm, was attached to the clip applicator. Then, the endoscope was reinserted, and the CWL was attached to the edge of the lesion.

**Fig. 3:**
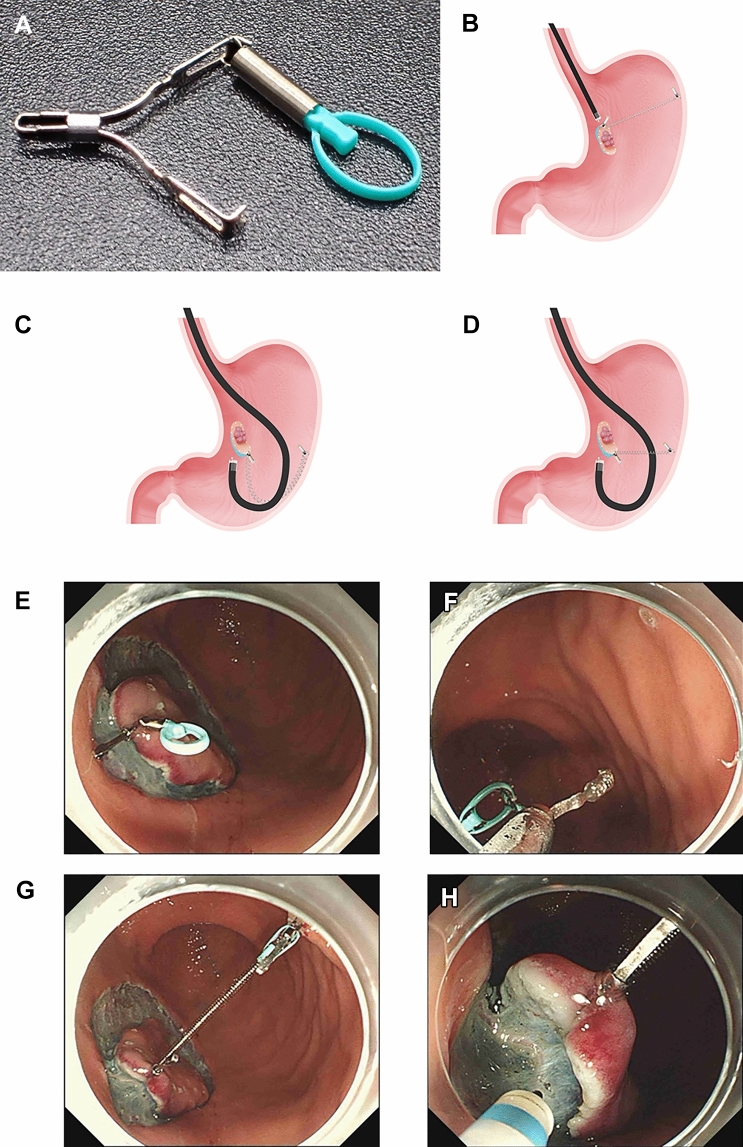
Spring-and-loop with clip (SLC)-assisted endoscopic submucosal dissection. **A** An SLC (Sakamoto-Osada clip; Zeon Medical, Tokyo, Japan) has a 5-mm-long spring and a 4-mm-long loop made of polyamide elastomers on one side of the clip claws. **B** In the forward endoscopic position, the endoscope rarely interferes with the SLC’s spring. **C** In the retroflexed endoscopic position, the endoscope can interfere with the SLC’s spring, resulting in overstretching and loss of elasticity. **D** The modified attachment method of SLC can prevent interference between the endoscope and the spring of SLC in the retroflexed endoscopic position. **E** After circumferential mucosal incision, the SLC is delivered via the endoscope’s accessory channel and attached to the lesion. **F** The regular clip hooks the loop of the SLC and anchors it to the gastric wall. **G** Vertical traction can be obtained regardless of the lesion location by selecting a site to anchor the SLC’s loop. **H** Vertical traction can expose the submucosa and provide effective tension on the dissection plane

SLC-ESD was performed using the modified attachment method of the SLC as previously reported (Fig. 3A–H) [7, 15, 16]. As much as possible, the forward endoscopic position was selected for submucosal dissection because it was less likely to cause interference between the endoscope and spring (Fig. 3B). Conversely, in the retroflexed endoscopic position, the endoscope can interfere with the spring of the SLC (Fig. 3C). Therefore, a modified attachment method was used to prevent this interference (Fig. 3D). After the circumferential mucosal incision, either the forward or retroflexed endoscopic position was selected for easier submucosal dissection. Next, the SLC was delivered through the accessory channel and attached to the edge of the lesion. Subsequently, another clip (ZP-CH, Zeon Medical) was used to anchor the loop portion of the SLC to a site that provided VT. If the spring shrunk after anchoring, the stomach was dilated via gas infusion to extend the spring. The anchor clip was removed from the gastric wall using forceps after resection, and the specimen, SLC, and anchor clip were extracted.

### Statistical analysis

The sample size was calculated according to the ESD time. In our pilot study, the mean ESD time was 21.4 min shorter in the SLC-ESD group than that in the CWL-ESD group, with a common standard deviation of 37.1 min for both groups. To ensure a power of 80% with a 5% two-sided error, we required 96 participants. Therefore, the targeted sample was 106 participants to permit a dropout rate of approximately 10%.

Multiple regression analysis was performed to identify the factors that affected the ESD time. VT was previously identified as an important factor for reducing ESD time [6, 7]. Previous reports revealed that gastric ESD can be technically challenging with a prolonged procedure based on the lesion location (upper and middle thirds of the stomach), specimen area (large), and the presence of submucosal fibrosis [1, 2, 17, 18]. Moreover, a lesion on the gravity side can increase the ESD time, as it causes visual field impairment attributable to halfway submergence. Therefore, the explanatory variables were as follows: VT, lesion location (upper and middle thirds of the stomach), specimen area, submucosal fibrosis, and gravity side. To approximate the ESD time and specimen area to a normal distribution, the common logarithmic conversion value was used for these factors.

All statistical analyses were performed using R version 4.3.1 (R Foundation for Statistical Computing, Vienna, Austria). Categorical variables were analyzed using Fisher’s exact test, and the data were presented as numbers and percentages. Continuous variables were reported as the median (interquartile range [IQR]), and differences between the groups were analyzed using the Mann–Whitney *U* test. In this study, all tests were two-sided, and differences between variables were considered statistically significant for *P* < 0.05.

## Results

### Patient characteristics

**Fig. 4:**
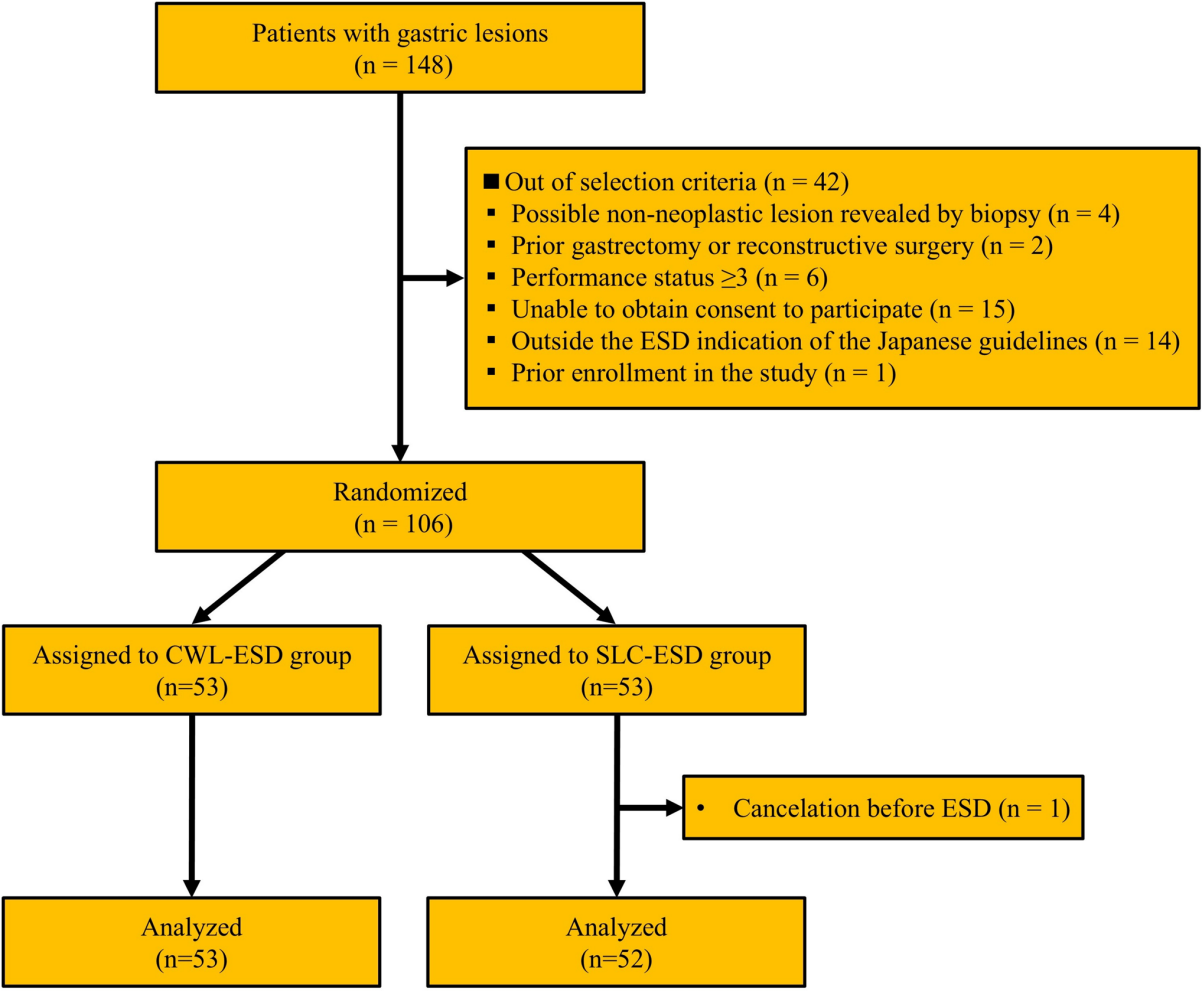
Flowchart of patient enrollment and group allocation. *ESD* endoscopic submucosal dissection; *CWL-ESD* clip-with-line–assisted ESD; *SLC-ESD* spring-and-loop with clip-assisted ESD

Figure 4 presents the flowchart of patient enrollment. Between August 2020 and April 2023, 106 patients were enrolled and randomly assigned to either the SLC-ESD or CWL-ESD group. One patient in the SLC-ESD group was excluded because the ESD was canceled preoperatively because of newly detected metastasis from another advanced cancer after enrollment. Except for this cancelation, all patients underwent treatment with the assigned traction device, which was successfully used without early abandonment. No cases required conversion to conventional methods. All patients assigned to the CWL-ESD group were included in the analysis. The baseline characteristics were well balanced between the two groups (Table 1).

**Table 1:** Baseline characteristics between the CWL-ESD and SLC-ESD groups

	CWL-ESD *n* = 53	SLC-ESD *n* = 52
Age, median [IQR]	74 [69–79]	74 [68–78]
Sex, female/male	16 (30.2)/37 (69.8)	15 (28.8)/37 (71.2)
*Lesion location*		
Upper third	8 (15.1)	11 (21.2)
Middle third	29 (54.7)	25 (48.0)
Lower third	16 (30.2)	16 (30.8)
*Lesion position*		
Greater curvature	7 (13.2)	5 (9.6)
Lesser curvature	20 (37.7)	26 (50.0)
Anterior wall	12 (22.6)	5 (9.6)
Posterior wall	14 (26.4)	16 (30.8)
*Morphology*		
Protruded (0–I, 0–IIa)	25 (47.2)	16 (30.8)
Flat (0–IIb)	5 (9.4)	1 (1.9)
Depressed (0–IIc, 0–III)	23 (43.4)	35 (67.3)

Values are expressed as *n* (%) unless otherwise indicated. *CWL-ESD* clip-with-line–assisted endoscopic submucosal dissection; *SLC-ESD* spring-and-loop with clip-assisted endoscopic submucosal dissection

### Procedure-related outcomes

Table 2 presents the procedure-related outcomes. One patient in the SLC-ESD group was diagnosed with a non-neoplastic lesion after ESD. This case was included in the analysis and was deemed an incomplete resection. Factors such as specimen size, specimen area, lesion size, histology, depth, presence of ulcer findings, and submucosal fibrosis did not significantly differ between the groups. The Shapiro–Wilk test and a quantile–quantile plot revealed a non-normal distribution of the ESD time in both groups. Moreover, the ESD time exhibited several outliers only in the SLC-ESD group. The median ESD time was significantly shorter in the SLC-ESD group than in the CWL-ESD group (26.0 min [IQR = 19.5–51.5] vs. 40.5 min [IQR = 26.0–62.2]; *P* = 0.015). The median dissection speed was also significantly faster in the SLC-ESD group (24.9 mm^2^/min [IQR] = 19.3–31.7 vs. 18.2 mm^2^/min [IQR] = 13.9–22.4; *P* = 0.001). En bloc resection was achieved without perforation in all patients in both groups. The complete resection rates were 98.1% and 96.2% in the SLC-ESD and CWL-ESD groups, respectively (*P* = 1.000).

**Table 2:** Procedure-related outcomes between the CWL-ESD and SLC-ESD groups

	CWL-ESD *n* = 53	SLC-ESD *n* = 52	*P*
*ESD time, min*			
Median [IQR]	40.5 [26.0–62.2]	26.0 [19.5–51.5]	0.015*
Mean (SD)	45.5 (24.1)	40.4 (41.3)	0.440
Range	13.0–101.8	8.2–273.6	
*Dissection speed, mm*^*2*^*/min*			
Median [IQR]	18.2 [13.9–22.4]	24.9 [19.3–31.7]	0.001*
Mean (SD)	19.2 (7.7)	26.5 (11.7)	< 0.001*
Range	2.9–37.6	6.9–53.8	
Specimen size, mm, median [IQR]	30 [27–40]	33 [27–41]	0.544
Specimen area, mm^2^, median [IQR]	635.9 [489.8–1036.2]	617.0 [508.7–1058.8]	0.813
Lesion size, mm, median [IQR]	15 [10–22]	12 [8–22]	0.211
Histology			0.072
Adenoma	7 (13.2)	6 (11.5)	
Differentiated adenocarcinoma	46 (86.8)	40 (76.9)	
Undifferentiated adenocarcinoma	0 (0)	5 (9.6)	
Non-neoplastic lesion	0 (0)	1 (1.9)	
Depth			0.361
Mucosa†	45 (84.9)	44 (84.6)	
Submucosa (< 500 μm)	2 (3.8)	5 (9.6)	
Submucosa (≥ 500 μm)	6 (11.3)	3 (5.8)	
Presence of ulcer findings	9 (17.0)	12 (23.1)	0.473
Submucosal fibrosis			0.873
F0	42 (79.2)	42 (80.8)	
F1	5 (9.4)	3 (5.8)	
F2	6 (11.3)	7 (13.5)	
Gravity side	6 (11.3)	6 (11.5)	1.000
En bloc resection	53 (100.0)	52 (100.0)	NA
Complete resection‡	51 (96.2)	51 (98.1)	1.000
*Adverse events*			
Post-ESD bleeding	3 (5.7)	0 (0.0)	0.243
Intraoperative perforation	0 (0)	0 (0)	NA
Delayed perforation	0 (0)	0 (0)	NA
Second knife	0 (0)	1 (1.9)	0.495
Multibending endoscope use	6 (11.3)	0 (0)	0.027*
*Traction device-related factors*			
Traction direction			< 0.001*
Proximal	28 (52.8)	0 (0)	
Diagonally proximal	6 (11.3)	0 (0)	
Vertical	6 (11.3)	52 (100)	
Diagonally distal	5 (9.4)	0 (0)	
Distal	8 (15.1)	0 (0)	
Attachment time, s, median [IQR]	99 [83–113]	88 [79–109]	0.236
Application, n, median [IQR]	1 [1–1]	1 [1–1]	0.435
Reattachment	4 (7.5)	2 (3.8)	0.678
Slip-off	1 (1.9)	0 (0)	1.000
Traction device-related damage to the specimen	1 (1.9)	0 (0)	1.000
Traction device breakage	0 (0)	0 (0)	NA
Successful removal of the anchor clip	NA	52 (100)	NA

*P < 0.05. Values are expressed as n (%) unless otherwise indicated. *CWL-ESD* clip-with-line–assisted endoscopic submucosal dissection; *SLC-ESD* spring-and-loop with clip-assisted endoscopic submucosal dissection; *IQR* interquartile range; *NA* not applicable. †This category includes intramucosal cancers, adenomas, and non-neoplastic lesions. ‡One case in the SLC-ESD group, pathologically diagnosed as a non-neoplastic lesion, was classified as an incomplete resection

The traction direction significantly differed between the groups (*P* < 0.001). VT was selected in all patients in the SLC-ESD group, compared with 11.3% of patients in the CWL-ESD group. Proximal traction (52.8%) was most commonly applied in the CWL-ESD group, while diagonally proximal traction was used in 11.3% of cases.

Post-ESD bleeding was observed in three patients in the CWL-ESD group but in none of the patients in the SLC-ESD group (*P* = 0.243). No perforations were observed in either group.

### Subgroup analysis

Table 3 presents the results of subgroup analyses for the ESD time according to the lesion location and size. The median ESD time was significantly shorter in the SLC-ESD group than in the CWL-ESD group for lesions located in the upper and middle thirds of the stomach and for lesions measuring ≤ 20 mm in size. However, the ESD time in the lower third lesions and lesions > 20 mm showed no significant differences.

**Table 3:** Subgroup analysis comparing the ESD time according to the lesion location and size

	CWL-ESD *n* = 53	SLC-ESD *n* = 52	*P*
*Lesion location*			
	*n* = 37	*n* = 36	
Upper and middle thirds	49.2 [32.7–62.5]	27.8 [20.9–53.3]	0.009*
	*n* = 16	*n* = 16	
Lower third	24.8 [17.9–32.3]	21.6 [16.2–45.3]	0.546
*Lesion size*			
	*n* = 39	*n* = 38	
≤ 20 mm	32.7 [24.5–49.6]	23.4 [17.9–28.9]	0.003*
	*n* = 14	*n* = 14	
> 20 mm	68.2 [48.0–93.9]	56.9 [46.3–75.4]	0.581

*P < 0.05. Values are expressed as the median [interquartile range] unless otherwise indicated. *CWL-ESD* clip-with-line–assisted endoscopic submucosal dissection; *SLC-ESD* spring-and-loop with clip-assisted endoscopic submucosal dissection

### Multivariate analysis

Table 4 presents the results of multiple regression analysis (F statistic; *P* < 0.001, adjusted *R*^2^ = 0.67, all variance inflation factors were < 1.04). VT, the lesion location, the specimen area, and the presence of submucosal fibrosis were independently associated with the ESD time.

**Table 4:** Multiple regression analysis of factors associated with the ESD time

Variables	β	95% CI	*P*
Vertical traction	− 0.289	− 0.434 to −0.145	< 0.001*
Lesion location (Upper and middle thirds)	0.271	0.114–0.428	< 0.001*
Specimen area	0.768	0.633–0.904	< 0.001*
Submucosal fibrosis	0.481	0.301–0.661	< 0.001*
Gravity side	0.197	− 0.027–0.422	0.084

*P < 0.05. β, regression coefficient; *CI* confidence interval; *ESD* endoscopic submucosal dissection

## Discussion

This study demonstrated that SLC-ESD reduced ESD time without increasing the risk of adverse events compared with CWL-ESD. VT was applied in all patients in the SLC-ESD group, while it was rarely applied in the CWL-ESD group. Multiple regression analysis indicated that VT was independently associated with a shorter ESD time. These results support our hypothesis that VT is the optimal direction in gastric ESD and that SLC-ESD with VT reduces ESD time compared with CWL-ESD.

From a physical perspective, traction is a force that can be represented using a vector characterized by size (strength) and direction. Although robot arm-assisted methods [19] and magnetic anchor methods [20] can control traction strength and direction, they have not yet been widely adopted in clinical practice. In contrast, CWL, sheath traction devices [21, 22], and internal traction devices (e.g., SLC, ring thread [23], rubber band and clip [24], and multi-loop traction devices [25]) are widely used in gastric ESD because of their reasonable costs and ease of use. An appropriate traction direction is essential for these devices because an inappropriate direction can complicate submucosal dissection, and changing direction after attachment requires a time-consuming procedure. However, the effect of traction direction on traction-assisted gastric ESD has not been adequately investigated.

Our results indicate that VT is the optimal direction in gastric ESD, as it effectively exposes the submucosa and provides adequate tension on the dissection plane. A previous multicenter RCT found that CWL-ESD did not reduce gastric ESD time compared with C-ESD in the total population, but it was linked to shorter ESD times for lesions located in the greater curvature of the upper and middle thirds of the stomach [6]. This may be explained by the fact that the traction direction of the CWL is limited to the cardia, making it anatomically difficult to provide VT unless the lesion is located in the greater curvature [26].

Although VT was applied in all cases in the SLC-ESD group, proximal traction was most frequently applied in the CWL-ESD group. Although proximal traction can cause the mucosal flap to fall toward the endoscope tip, thereby impeding access to the submucosa, it can be effective when the endoscope tip approaches the lesion parallel to the gastrointestinal wall because the tip can easily access the submucosa without VT [27]. Indeed, CWL-ESD provides proximal traction in esophageal ESD, effectively reducing ESD time compared with C-ESD, as shown in a recent multicenter RCT [28]. However, proximal traction is not always effective in gastric ESD owing to the large and angulated stomach lumen, which can make it difficult to achieve a parallel approach to the lesion. Additionally, distal or diagonally distal traction, often used with a retroflexed endoscopic position in CWL-ESD, can cause the dissection plane to fall distally as submucosal dissection advances, complicating the procedure. Contrarily, diagonally proximal traction can have similar efficacy to VT because it has a vertical component and does not cause the dissection plane to fall distally. However, diagonally proximal traction was not commonly obtained in the CWL-ESD group.

In terms of cost-effectiveness, an SLC is approximately five-fold more expensive than a CWL (approximately ¥5000 vs. ¥1000 [$33 and $7, respectively, at an exchange rate of ¥150 = $1]). Therefore, CWL-ESD should be considered for lesions located in the greater curvature, where CWL-ESD can achieve VT.

Although SLC enables controlled traction direction regardless of lesion location, a learning curve may exist because of the need for precise anchor clip placement. However, by marking the planned anchoring site before SLC attachment, accurate anchoring may be achieved. A previous study demonstrated that endoscopists familiar with hemoclips could use the modified SLC attachment method without marked difficulty, even during its initial adoption [16].

According to the results of subgroup analyses, SLC-ESD was not associated with a shorter ESD time for lesions located in the lower third of the stomach or for those >20 mm in size, in line with our previous findings [7, 8, 15]. This might be attributable to the difficulty in maintaining the strength of traction. The lumen of the lower third of the stomach is relatively narrow, making it difficult to obtain sufficient spring extension. Moreover, if the lesion is large, the distance between the SLC on the lesion and the anchor site is smaller, causing the spring to shrink as submucosal dissection advances. In such situations, traction devices capable of both controlling traction direction and increasing traction strength, such as multi-loop traction device [29], adaptive multipolar traction system [30], rubber band and clip [24], or CWL with a clip pulley [31, 32], may help address this limitation. In particular, a rubber band and clip or a CWL with a clip pulley is less expensive than an SLC, and recent advances in reopenable clips have facilitated easier attachment of rubber bands or pulleys.

Although an SLC is currently available in a few countries, other self-producible internal traction devices that can control the traction direction, such as a ring thread [23] or a rubber band and clip [24], could be alternatives to the SLC. However, these devices might be less adjustable for the large stomach lumen because of the lower elasticity of the material. Therefore, the loop or band size should be selected appropriately according to the size of the lumen when using these devices. In addition to the abovementioned internal traction devices, a CWL with a clip pulley (per-oral traction device) [31, 32] can be self-produced and control the traction direction.

This study had several limitations. First, the operator could not be blinded, leading to possible performance bias. However, the ESD time and dissection speed were improved in the CWL-ESD group compared with those in our previous study [8]. Second, a single operator with sufficient experience in both CWL-ESD and SLC-ESD performed all procedures. A multicenter RCT involving endoscopists with various skill levels should be conducted. Third, this study did not compare VT with other directions because the intervention focused specifically on the use of a traction device. Based on our findings, although VT appears to be the optimal traction direction in gastric ESD, another traction direction might be better than VT. Further studies comparing traction directions are needed to clarify the optimal traction direction. Fourth, the ESD time was compared using a nonparametric test with median values, although the sample size was calculated using the mean values. However, the ESD time was not normally distributed in both groups; thus, a nonparametric test with median values is appropriate.

In conclusion, our findings indicate that SLC allows for controlled traction direction regardless of lesion location. SLC may be more effective than CWL, particularly for relatively smaller lesions or lesions located in larger lumen areas (e.g., upper- and middle-third of the stomach) because it allows for controlled traction direction with sufficient force. The ability to selectively control the traction direction may improve the effectiveness of traction-assisted gastric ESD.

## Supplementary Information

Below is the link to the electronic supplementary material.

Video 1. Differences between clip-with-line–assisted endoscopic submucosal dissection and spring-and-loop with clip-assisted endoscopic submucosal dissection in the stomach. Supplementary file1 (WMV 92561 kb)
